# Identification of Novel Genetic Variants and Food Intake Factors Associated with Type 2 Diabetes in South Korean Adults, Using an Illness–Death Model

**DOI:** 10.3390/ijms26062597

**Published:** 2025-03-13

**Authors:** Jeongmin Oh, Junho Cha, Sungkyoung Choi

**Affiliations:** 1 Department of Applied Mathematics, College of Science and Convergence Technology, Hanyang University, 55 Hanyang-daehak-ro, Sangnok-gu, Ansan 15588, Republic of Korea; ojm0027@hanyang.ac.kr; 2 Department of Applied Artificial Intelligence, College of Computing, Hanyang University, 55 Hanyang-daehak-ro, Sangnok-gu, Ansan 15588, Republic of Korea; chajunho822@hanyang.ac.kr; 3 Department of Mathematical Data Science, College of Science and Convergence Technology, Hanyang University, 55 Hanyang-daehak-ro, Sangnok-gu, Ansan 15588, Republic of Korea

**Keywords:** type 2 diabetes, prediabetes, multi-state illness–death model, Cox proportional hazards model, Korean genome and epidemiology study, genome-wide association study

## Abstract

Type 2 diabetes (T2D) is a prevalent chronic disease in the Korean population, influenced by lifestyle, dietary habits, and genetics. This study aimed to identify the effects of food intake and genetic factors on T2D progression in Korean adults using a multi-state illness-death model. We analyzed three transition models: normal glucose tolerance (NGT) to prediabetes (PD), NGT to T2D, and PD to T2D. We first identified dietary patterns significantly associated with each transition, using multivariate Cox proportional hazards models. Then, we assessed the impact of single-nucleotide polymorphisms (SNPs) on each transition, incorporating these dietary patterns as covariates. Our analysis revealed significant associations between the identified dietary patterns and the risk of PD and T2D incidence among individuals with NGT. We also identified novel genetic variants associated with disease progression: two SNPs (*rs4607517* in Glucokinase [*GCK*] and *rs758982* in Calcium/Calmodulin-Dependent Protein Kinase II Beta [*CAMK2B*]) in the NGT to PD model, and eight SNPs in the NGT to T2D model, including variants in the Zinc Finger Protein 106 (*ZNF106*), PTOV1 Extended AT-Hook Containing Adaptor Protein (*PTOV1*), Proprotein Convertase Subtilisin/Kexin Type 2 (*PCSK2*), Forkhead Box D2 (*FOXD2*), Solute Carrier Family 38 Member 7 (*SLC38A7*), and Neuronal Growth Regulator 1 (*NEGR1*) genes. Functional annotation analysis using ANNOVAR revealed that *rs4607517* (*GCK*) and *rs59595912* (*PTOV1*) exhibited high Combined Annotation-Dependent Depletion (CADD) and Deleterious Annotation of Genetic Variants using Neural Networks (DANN) scores, suggesting potential pathogenicity and providing a functional basis for their association with T2D progression. Integrating dietary and genetic factors with a multi-state model, this comprehensive approach offers valuable insights into T2D development and highlights potential targets for prevention and personalized interventions.

## 1. Introduction

Type 2 diabetes (T2D) has reached pandemic proportions, affecting 537 million adults globally in 2023, with the number of cases projected to surpass 783 million by 2045, due to aging populations and urbanization [1]. In South Korea, T2D prevalence has surged, impacting 6 million adults (14.4% prevalence), while prediabetes (PD) affects 15.83 million individuals, placing over 40% of the population at risk [2]. T2D significantly elevates mortality from cardiovascular disease, stroke, and dementia, with annual healthcare costs exceeding $966 billion globally [3,4,5,6]. These trends underscore the urgency of understanding the genetic and environmental drivers of T2D progression [7].

T2D arises from a complex interplay between insulin resistance and β-cell dysfunction [8,9,10]. In the early stages, compensatory hyperinsulinemia maintains normoglycemia, despite insulin resistance in the liver, muscle, and adipose tissue [11,12,13,14,15]. The transition from normal glucose tolerance (NGT) to PD is marked by elevated fasting glucose (100–125 mg/dL) and postprandial hyperglycemia, driven primarily by insulin resistance [8,16]. Over time, chronic insulin resistance overwhelms β-cells, leading to progressive dysfunction, impaired insulin secretion, and overt hyperglycemia [9,14]. This progression highlights distinct mechanisms at different disease stages, necessitating stage-specific interventions.

Genome-wide association studies (GWASs) have identified over 400 loci influencing T2D risk, many of which regulate insulin secretion (Glucokinase [*GCK*], Transcription Factor 7-Like 2 [*TCF7L2*]) or insulin sensitivity (Peroxisome Proliferator-Activated Receptor Gamma [*PPARG*], Insulin Receptor Substrate 1 [*IRS1*]) [17,18,19,20]. Single-nucleotide polymorphisms (SNPs)—single-base DNA variations altering gene function—play critical roles. Population-specific variants, such as 19 SNPs identified in Korean cohorts, emphasize the need for ethnically diverse studies [21]. Functional annotation reveals that SNPs like Calcium/Calmodulin-Dependent Protein Kinase II Beta (*CAMK2B*) *rs758982* may impair β-cell Ca^2+^ signaling, exacerbating insulin secretory defects. Furthermore, Ohn et al. (2016) discovered that the SNP *rs4607517*, located in the Glucokinase (*GCK*) gene, disrupts glucose sensing in β-cells, elevating fasting glucose in Koreans [22].

Environmental factors, particularly lifestyle habits, play a significant role in the onset of T2D. These include dietary patterns, nutrient consumption, energy intake, physical activity, smoking status, and alcohol intake [16,23]. Notably, increases in energy intake and decreases in energy expenditure often lead to obesity. This obese state frequently progresses to insulin resistance, making it a significant risk factor for the onset of T2D [24]. T2D can exhibit an increased incidence due to the synergistic effects of genetic and lifestyle factors. Identifying the effect of the relationship between genetic and lifestyle factors on disease can be used to predict the risk of development of the disease in subjects with genetic risk factors, and to prevent disease through making changes to risky lifestyles [25]. Despite these insights, most studies have focused on isolated nutrients or single environmental factors, rather than comprehensive dietary patterns or lifestyle behaviors, limiting the translational applicability of the findings. A more integrative approach is needed in order to better understand the complex interactions between genetic predisposition and environmental exposures in the development of T2D.

Traditional epidemiological approaches often fail to capture the dynamic and progressive nature of T2D, particularly the transitions between NGT, PD, and T2D. These methods typically focus on static associations, overlooking the stage-specific risk factors influencing disease progression. The multi-state illness–death model addresses these limitations by quantifying transition-specific risks and identifying modifiable factors at critical junctures [26,27,28,29,30]. For instance, Minooee et al. (2019) demonstrated that low testosterone levels significantly increased the risk of transitioning from NGT to PD (HR = 0.94), but not from PD to T2D, highlighting the stage-specific nature of risk factors [29]. Similarly, Yerramalla et al. (2020) showed that physical activity reduced the incidence of T2D (HR = 0.85), emphasizing its protective role in disease progression [30]. By modeling transitions between distinct disease states, the multi-state illness–death model provides a nuanced understanding of T2D development and enables targeted interventions for its prevention and management.

This study utilizes Korean Genome and Epidemiology Study (KoGES) data, from a large-scale, population-based cohort designed to investigate the genetic and environmental determinants of chronic diseases, including T2D [31]. The KoGES provides comprehensive longitudinal data on lifestyle, dietary habits, clinical measurements, and genomic profiles, making it a valuable resource for studying complex gene–environment interactions. Among the various subcohorts within the KoGES, we focused on the Ansan/Ansung cohort, which includes 10,030 participants, aged 40–69 years, from urban (Ansan) and rural (Ansung) areas of South Korea. The Ansan/Ansung cohort provides several distinct advantages for investigating the progression of type 2 diabetes (T2D). First, its longitudinal design includes biennial follow-ups conducted from 2001 to 2016, allowing for repeated measurements of dietary intake, clinical parameters, and genomic data over time. Second, genome-wide genetic data were obtained using the Korea Biobank Array (KCHIP), which includes over 800,000 SNPs tailored explicitly to the Korean population, enabling a comprehensive analysis of genetic risk factors [32]. Third, dietary intake was assessed using a validated semi-quantitative food frequency questionnaire (SQ-FFQ), facilitating detailed examinations of dietary patterns and their associations with disease progression. Finally, the cohort’s ethnic specificity captures unique gene–environment interactions in the Korean population, an under-represented group in global GWASs. These features make the Ansan/Ansung cohort an invaluable resource for studying T2D progression and understanding its complex genetic and environmental determinants. For this study, we analyzed data from 4126 participants who met the inclusion criteria of having NGT or PD at baseline and complete dietary and genetic data available for analysis. By applying a multi-state illness–death model to this cohort, we aimed to identify stage-specific genetic and dietary factors influencing the transitions between NGT, PD, and T2D. This approach addresses gaps in previous research by examining T2D progression in an ethnically specific population.

## 2. Results

### 2.1. Demographic and Lifestyle Characteristics

At baseline, the mean age of participants was 51.0 ± 8.4 years, with a mean body mass index (BMI) of 24.5 ± 3.0 kg/m^2^. The study population consisted of slightly more females (52.9%) than males. The majority of participants were from the Ansan area (56.3%), while 43.7% were from the Ansung area. Most participants (68.5%) reported engaging in regular physical activity.

Regarding lifestyle factors, 48.8% of participants were current alcohol consumers, while 23.3% were current smokers. The participants’ educational backgrounds varied, with 14.5% having attained a college or higher level of education, 57.2% with middle/high school education, and 28.3% with elementary school education or less. In terms of household income, 19.8% reported earning 3 million Korean won or more per month, while 30.1% earned less than 1 million Korean won per month.

Dietary intake patterns were assessed using median weekly servings. The median (interquartile range) intake for various food groups was as follows: fruit—12.3 (4.4–14.1) servings/week, vegetables—26.6 (14.6–33.4) servings/week, red meat—2.8 (1.1–3.6) servings/week, white meat—0.8 (0.2–0.9) servings/week, grains—25.6 (22.7–26.4) servings/week, fish—8.8 (3.7–11.8) servings/week, and dairy products—5.2 (0.8–7.8) servings/week.

This revised description provides a more comprehensive and accurate representation of the baseline characteristics of the study population, aligning closely with the data presented in Supplemental Table S1.

### 2.2. Association Between Food Intake and T2D

During the 14-year follow-up period, 1706 cases of PD were reported among 4126 participants. A total of 423 cases of T2D were observed, with 151 cases transitioning directly from normal glucose tolerance (NGT) to T2D (T_02_), and 272 cases progressing from PD to T2D (T_12_). Table 1 shows the results of the multivariate Cox proportional hazards model, which included covariates such as age (continuous), BMI (continuous), sex (categorical), residential area (categorical), physical activity (categorical), alcohol intake (categorical), smoking status (categorical), education level (categorical), household income (categorical), and cumulative averages of food intake categorized into tertiles (fruit, vegetables, red meat, white meat, grains, fish, and dairy).

In the NGT to PD transition, higher consumption of red meat and white meat was associated with an increased risk of PD. Comparing the highest- and the lowest-intake categories, the multivariate hazard ratios (HRs) (95% confidence interval, CIs) were 1.41 (1.08 to 1.85, *p* = 0.012) for red meat and 1.61 (1.26 to 2.07, *p* < 0.001) for white meat. Conversely, a higher intake of fruit and dairy products was associated with a lower risk of PD (fruit: HR = 0.18, 95% CI = 0.14 to 0.24, *p* < 0.001; dairy: HR = 0.62, 95% CI = 0.49 to 0.78, *p* < 0.001).

For the NGT to T2D transition, high fish intake was associated with a 1.62 times higher risk of T2D than low fish intake (HR = 1.62, 95% CI = 1.00 to 2.62, *p* = 0.048). In contrast, higher consumption of fruit and vegetables was associated with a lower risk of T2D (fruit: HR = 0.60, 95% CI = 0.39 to 0.93, *p* = 0.022; vegetable: HR = 0.63, 95% CI = 0.40 to 1.00, *p* = 0.048).

In the PD to T2D transition, no significant associations were observed between food intake and T2D risk. Additionally, the consumption of grains did not show significant associations with the risk of PD or T2D in any of the transition models.

### 2.3. Association Between SNP and T2D

After quality control, 537,149 SNPs and 4126 participants were included in the genome-wide association study (GWAS) analysis. Significant food variables were included as covariates in the Cox proportional hazards model, to identify SNPs associated with the risk of PD and T2D in each transition model. The NGT to PD model was adjusted for age, sex, residential area, physical activity, alcohol intake, smoking status, education level, household income, BMI, and intake of fruit, red meat, white meat, and dairy. The NGT to T2D model was adjusted for the epidemiological variables, plus fruit, vegetable, and fish intake. The PD to T2D model included only epidemiological variables as covariates.

The overall statistical associations of genetic variants with T2D are shown as Manhattan plots (Figure S1) using Cox regression. Based on the false discovery rate (FDR) adjusted *p*-value (*q*-value) < 0.05 criterion, two SNPs showed significant associations in the NGT to PD model, and eight SNPs showed significantly associations in the NGT to T2D model. In contrast, no significant SNPs were identified in the PD to T2D model. Table 2 shows the characteristics of these novel variants. In the model for the transition from NGT to PD, *rs4607517* in the *GCK* gene was reported to have the highest significance, with an HR of 1.27 (95% CI = 1.17 to 1.37) and a *p*-value of 1.37 × 10^−9^. Additionally, *rs758982* in the *CAMK2B* gene showed a comparable effect size, with an HR of 1.27 (95% CI = 1.18 to 1.38), indicating a significant role in the progression from NGT to PD. In the model for the transition from NGT to T2D, eight SNPs were associated with an increased risk of T2D development, with HRs ranging from 1.88 to 4.31. The identified SNPs were located in or near diverse genes: *ZNF106* (*rs145386384*), *PTOV1* (rs59595912), *LOC105374834* (*rs7575023*), *LINC00557* (*rs35566993*), *PCSK2* (*rs11698919*), *FOXD2* (*rs59813747*), *SLC38A7* (*rs4784964*), and *NEGR1* (*rs147467153*). Notably, the *NEGR1* variant (*rs147467153*) exhibited the strongest association, with individuals carrying this variant having a 4.31-fold higher risk of developing T2D compared to non-carriers. The *ZNF106* variant (*rs145386384*) also showed a substantial effect, with a 3.77-fold increased risk.

**Figure 1:**
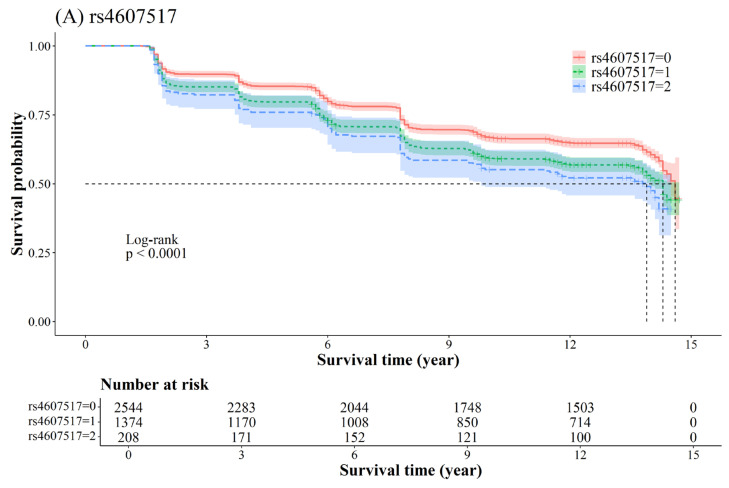

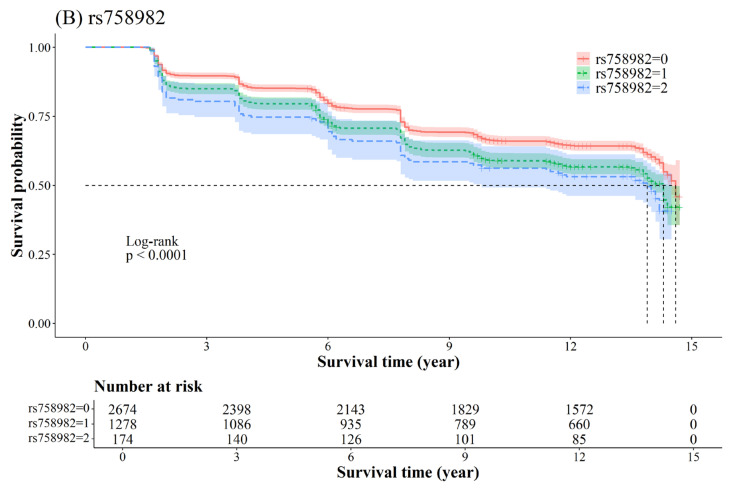
Kaplan–Meier survival curves for prediabetes risk based on SNP allele counts. Kaplan–Meier survival curves illustrate cumulative probability of transitioning from normal glucose tolerance (NGT) to prediabetes (PD) over a 14-year follow-up period, based on allele counts of two SNPs: *rs4607517* in *GCK* gene and *rs758982* in *CAMK2B* gene. Separate curves are presented for individuals with 0 (upper red line), 1 (middle green dashed line), or 2 (lower blue line) risk alleles. The horizontal dashed line at Survival Probability = 0.5 represents the point where the survival probability is 50%, indicating the time at which the probability drops to this level for each genotype group. Log-rank tests assessed statistical differences between groups (*p* < 0.0001), demonstrating significant association between allele count and PD risk. (**A**) Survival curve for *rs4607517*. (**B**) Survival curve for *rs758982*.

Kaplan–Meier analysis revealed notable variations in disease progression, based on allele counts for specific genetic variants. For the transition from NGT to PD, Figure 1 shows the cumulative probability of PD development in relation to the number of alleles present for *rs4607517* (*GCK* gene) and *rs758982* (*CAMK2B* gene). Individuals carrying two alleles of these variants exhibited a significantly higher likelihood of developing PD than those without any alleles (log-rank *p* < 0.0001). Similarly, Supplemental Figure S2 presents Kaplan–Meier curves illustrating the cumulative probability of T2D onset from NGT. These survival curves also demonstrate statistically significant differences among the various genotypes (log-rank *p* < 0.0001), suggesting that allele count plays a crucial role in determining the risk of T2D development from NGT. Thus, we can conclude the potential impact of specific genetic variants on the progression of T2D, with higher-risk alleles associated with an increased likelihood of disease development.

A further analysis was conducted on chromosome 7 to investigate the genetic associations, focusing on a specific region containing 153 SNPs (from position 43,835,668 to 44,635,668 base pair). The regional plot in Supplemental Figure S3 provides a comprehensive view of this chromosomal area. The blue line in the plot represents recombination rates, estimated using data from the hg19/1000 genomes in the November 2014 Asian population. On the left side of the plot, a color scheme indicates the strength of the linkage disequilibrium (*r*^2^) between *rs4607517* (represented by a purple diamond) and surrounding SNPs. In the NGT to PD model, *rs4607517* showed the strongest association, with the lowest adjusted *p*-value (*q*-value = 0.0006). Notably, *rs758982* in the *CAMK2B* gene demonstrated a high linkage disequilibrium with *rs4607517*, with an *r*^2^ value exceeding 0.8. This suggests a strong genetic correlation between these two variants. In contrast, the remaining 133 SNPs in the region showed a weak linkage disequilibrium with *rs4607517*, all having *r*^2^ values below 0.2. Interestingly, *rs4607517* and *rs758982* exhibited identical hazard ratios (HR = 1.27) for PD development.

### 2.4. Functional Annotation

To evaluate the potential impact of the 10 identified SNPs on PD and T2D risk, we conducted a functional annotation analysis using ANNOVAR (version 2018Apr16, https://annovar.openbioinformatics.org/en/latest/), based on the reference genome hg19/GRCh37. The analysis employed two key scoring systems: the Combined Annotation-Dependent Depletion (CADD) score and the Deleterious Annotation of Genetic Variants using Neural Networks (DANN) score. The CADD score, scaled using the Phil’s Read Editor (PHRED), provides a measure of deleteriousness, with higher scores indicating potentially more harmful variants. A score of 10 represents the top 10% of deleterious variants, while a score of 20 indicates the top 1%. The DANN score ranges from 0 to 1, with scores closer to 1 suggesting a higher likelihood of pathogenicity.

Among the ten significant SNPs, two SNPs had CADD scores above the threshold of 12.37, indicating deleterious variants [33], and had DANN scores greater than 0.8, suggesting pathogenic variants. In the NGT to PD transition model, *rs4607517* in the *GCK* gene (chromosome 7) showed a CADD score of 14.470 and a DANN score of 0.877 (Table 3). In the NGT to T2D transition model, *rs59813747* in the *FOXD2* gene (chromosome 1) demonstrated a CADD score of 12.890 and a DANN score of 0.994. The remaining variants showed varying degrees of potential impact, with CADD scores ranging from 0.025 to 8.908 and DANN scores ranging from 0.318 to 0.802. Notably, *rs4784964* in the *SLC38A7* gene (chromosome 16), also identified in the NGT to T2D model, had a relatively high DANN score of 0.802, despite a lower CADD score of 4.847.

## 3. Discussion

We investigated the effects of dietary patterns and genetic markers on the progression of prediabetes (PD) and type 2 diabetes (T2D) in a Korean population using a multi-state illness–death model. This approach allowed us to analyze stage-specific transitions between normal glucose tolerance (NGT), PD, and T2D, providing valuable insights into the influence of food intake and SNPs on the stages of T2D progression.

### 3.1. Food Intake Factors

In this study, our results showed significant associations between specific dietary patterns and the incidence of PD and T2D. In the transition from NGT to PD, we observed an inverse association between the intake of fruit and dairy products and PD incidence, while red and white meat consumption showed a positive association. For the NGT to T2D transition, lower fruit and vegetable intake and higher fish consumption were associated with an increased risk of T2D.

These results align with previous epidemiological studies investigating the relationship between food consumption and the risk of developing PD and T2D. Previous studies have demonstrated inverse associations between fruit and vegetable intake and the risk of PD and T2D [34,35,36,37,38]. Furthermore, Safabakhsh et al. reported that higher consumption of total fruits and vegetables was associated with a lower risk of PD [39]. Similarly, a Swedish study found inverse associations between higher tertiles of total fruit and vegetable intake and T2D risk, and between higher fruit intake and PD risk in men [40]. The protective effect of fruits and vegetables against PD could be attributed to their high content of antioxidants and fiber, which can improve glucose tolerance, regulate glucose levels, and enhance insulin secretion [41,42,43]. Conversely, our finding of an association between increased fish intake and higher T2D risk is supported by previous cohort studies [44,45,46]. This relationship may be explained by the generation of advanced glycation end products (AGEs) during high-temperature cooking methods for fish, such as grilling or frying, which are known to contribute to insulin resistance [47]. Several cohort studies have reported that a higher red and white meat intake increases the risk of T2D development [48,49,50,51]. A cohort study in the United States indicated a significant association between increased red meat and poultry intake and T2D development [52]. Isanejad et al. showed that higher poultry intake may contribute to increased T2D risk [53], while the Nurses’ Health cohort study found that intake of red and processed meat was positively associated with T2D incidence in females [54]. Higher levels of saturated fat in meat have been associated with an increased risk of glucose intolerance and T2D [55,56]. While an inverse association between dairy product intake and T2D risk has been reported in previous epidemiological studies [57,58,59,60], few have explored its association with PD risk. The Rotterdam study showed that high-fat yogurt was inversely associated with lower PD risk and longitudinal insulin resistance [61]. The Framingham Offspring study found beneficial associations between total dairy consumption and PD development [62]. Dairy products containing vitamin D and calcium may optimize glucose metabolism and affect glucose homeostasis [63].

These findings suggest that food intake can be a protective or risk factor for developing PD and T2D. Further studies are needed to examine the benefits of individual food components and their specific roles in T2D disorders.

### 3.2. Genetic Variants

Our comprehensive analysis of genetic variants associated with PD and T2D progression revealed several significant SNPs across different transition stages. We identified 10 SNPs using a multi-state illness–death model, and assessed their potential risk using genome-wide functional prediction scores (CADD and DANN). Notably, two SNPs (*rs4607571* and *rs59813747*) were classified as deleterious, with CADD scores > 12.37 and DANN scores > 0.8, suggesting their potential pathogenicity.

In the transition from NGT to PD, we observed significant changes in *rs4607517* (*GCK* gene) and *rs758982* (*CAMK2B* gene). The *GCK* gene, encoding glucokinase, is crucial in regulating insulin secretion and glucose sensing in pancreatic beta-cells [64,65,66]. Mutations in the *GCK* gene have been associated with mild but stable elevations in plasma glucose levels, leading to glucose-sensing defects [64,65]. The *CAMK2B* gene, encoding Ca^2+^/calmodulin-dependent protein kinase IIβ, interacts with Ca^2+^-independent phospholipase A2 (iPLA2β) to form a signaling complex in β-cells, potentially influencing insulin secretion in T2D [67]

The transition from NGT to T2D was marked by significant changes in eight SNPs, including *rs59813747* (*FOXD2* gene), *rs147467153* (*NEGR1* gene), *rs145386384* (*ZNF106* gene), *rs4784964* (*SLC38A7* gene), *rs59595912* (*PTOV1* gene), *rs11698919* (*PCSK2* gene), and two intergenic variants (*rs35566993* and *rs7575023*) near *LINC00557* and *LOC105374834*. Of particular interest are the *SLC38A7*, *PCSK2*, and *NEGR1* genes, which have been implicated in various aspects of glucose metabolism and T2D risk. The *SLC38A7* gene, encoding a Na^+^-coupled glutamine transporter, is linked to the mTORC1 signaling pathway, which is crucial in nutrient sensing and insulin resistance [68,69]. mTORC1 hyperactivation can lead to insulin resistance through multiple mechanisms, including the phosphorylation of IRS-1 and the regulation of insulin signaling via Grb10 [70,71,72,73]. The *PCSK2* gene, encoding prohormone convertase 2, is involved in proinsulin processing, and interacts with the *TCF7L2* gene, a gene strongly associated with T2D risk [74,75,76]. TCF7L2 is a crucial regulator of proinsulin synthesis, processing, and clearance. Its influence extends to insulin resistance and diabetes, as it interacts with and controls the *PCSK2* gene [76]. *PCSK2* variants have been consistently reported as diabetes susceptibility genes in previous GWASs [77,78,79,80]. The *NEGR1* gene, which encodes neuronal growth regulator 1, has been associated with abnormal fat accumulation and elevated serum glucose and insulin levels in animal models [81]. Several GWASs have identified *NEGR1* variants as risk factors for obesity and T2D susceptibility [82,83,84,85,86,87].

Our results provide valuable insights into the genetic architecture of PD and T2D progression. However, further research is necessary to elucidate the precise mechanisms by which these genetic variants influence disease progression. Future studies should focus on the functional characterization of these variants and their potential interactions with environmental factors. Additionally, longitudinal studies tracking individuals with these genetic variants could provide insights into their long-term impact on disease progression and treatment response. In conclusion, our findings contribute to the growing body of evidence supporting the role of genetic factors in diabetes progression. Identifying stage-specific genetic associations may affect personalized prevention strategies and targeted interventions in managing PD and T2D.

### 3.3. Strengths and Limitations

Our study possesses several notable strengths that enhance its significance in diabetes research. Firstly, it is a population-based prospective study with a substantial follow-up period of 14 years, incorporating genome-wide data and direct repeated measurements of T2D states. This longitudinal design allows for a robust assessment of disease progression and the identification of risk factors over time.

The key strength of our study lies in its integrative approach, combining dietary and genetic influences on T2D risk. We not only investigated the impact of food intake, but also conducted a GWAS, which led to the discovery of 10 novel genetic variants potentially contributing to PD and T2D risk. This comprehensive examination of both environmental and genetic factors provides a more holistic understanding of the complex etiology of T2D. Furthermore, utilizing the multi-state illness–death model represents a methodological advancement in diabetes research. This advanced statistical modeling approach allows for a nuanced analysis of T2D progression by considering three distinct states: NGT, PD, and T2D. By employing this model, we gain deeper insights into the transitional processes between these states, offering a more accurate representation of the disease trajectory.

However, our study also has several limitations that warrant consideration. Firstly, individuals transitioning from PD to T2D may have already modified their dietary and lifestyle habits, due to awareness of their prediabetic status. This self-regulation could potentially introduce bias in analyzing the association between food intake and T2D risk. Secondly, residual confounding may still exist, despite extensive adjustment for potential confounding factors. Our study focused on the effects of food intake as broad categories, rather than individual food components. Given the complexity of dietary patterns, a more detailed categorization could provide deeper insights into specific nutritional factors influencing T2D progression. Thirdly, there is an imbalance in the number of case and control subjects for the NGT to T2D model and PD to T2D model, which may limit the statistical power to identify significant common variants. Replicating our findings in other cohorts is necessary to validate and extend our results. Lastly, one limitation of this study is that we did not conduct separate analyses for the cohort’s urban (Ansan) and rural (Ansung) subgroups. While residential area was included as a covariate in our models, environmental or lifestyle factors unique to each region may influence disease progression differently. Future studies with larger sample sizes or stratified analyses may provide additional insights into these potential urban–rural differences. Despite these limitations, our study contributes valuable insights into the complex interplay of dietary and genetic factors in T2D development and progression. Future research should address these limitations by incorporating more detailed dietary assessments, considering additional confounding factors, and replicating findings in more extensive, diverse cohorts.

## 4. Materials and Methods

### 4.1. Study Population

This study utilized the Ansan/Ansung combined longitudinal study data collected by the KoGES under the Korea Disease Control and Prevention Agency (KDCA), and used data from 2001 to 2002 (baseline survey) through to 2015 to 2016 (seventh follow-up survey) [31]. The Ansan/Ansung combined longitudinal study targeted a general population of males and females aged 40 to 69, collecting data through various surveys related to lifestyle factors such as health status, disease history, physical activity, and dietary habits. This cohort study followed participants biennially for repeated follow-ups. The study design and procedure details have been described previously [88,89].

**Figure 2:**
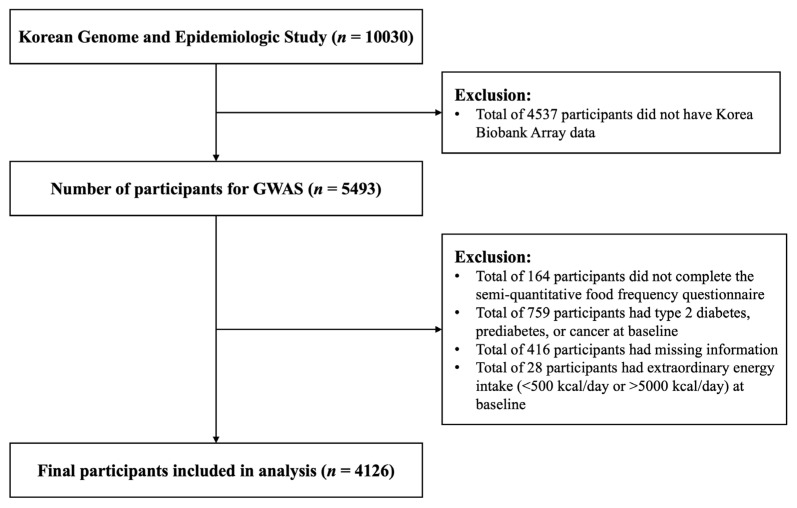
Flow diagram of participant inclusion and exclusion criteria. This figure outlines inclusion and exclusion criteria applied to participants from Ansan/Ansung cohort in Korean Genome and Epidemiology Study (KoGES). Diagram details participant selection from baseline through follow-up surveys, highlighting exclusions due to incomplete data, pre-existing conditions, or extreme energy intake levels, providing clear overview of how final study sample of 4126 participants was derived.

This study involved 10,030 participants, and included the genetic data of 5943 participants at baseline (2001–2002) [32]. Of the 5493 participants in the baseline survey, 164 participants who did not complete the food frequency questionnaire, 759 participants who had T2D, PD, or cancer at baseline, 416 participants who had missing information, and 28 participants who had extraordinary energy intake (<500 kcal/day or >5000 kcal/day) at baseline were excluded from this study. Finally, 4126 participants participated in this study (Figure 2). The study protocol was approved by the Institutional Review Board of Hanyang University (IRB no. HYUIRB-202402-010), and all participants provided written informed consent to participate in the study.

### 4.2. General Characteristics and Anthropometric Measurements

The participants were questioned by trained interviewers about their demographics, medical history, lifestyles, and physical examinations [88,89]. Questionnaires were conducted to obtain general characteristics, including age (years), sex, residential area (Ansan and Ansung), educational level (elementary (≤6 years), middle/high (7 to 12 years), and college/higher (>12 years)), household income (<1 million Korean won, 1 to 2 million Korean won, 2 to 3 million Korean won, and ≥3 million Korean won per month), alcohol intake (never, past, and current), and smoking status (never, past, and current). Physical activity was classified into ‘YES’ and ‘NO’ based on a high or moderate number of hours of physical activity during the day. The weight (kg) and height (cm) of the participants were measured with them wearing light clothing and no shoes, and BMI was calculated as weight divided by height squared (kg/m^2^).

### 4.3. Definition of Type 2 Diabetes and Prediabetes

T2D and PD were defined based on the diagnostic criteria established by the American Diabetes Association (ADA) [90]. Specifically, T2D was diagnosed as a fasting plasma glucose (FPG) concentration ≥ 126 mg/dL (7.0 mmol/L), the use of diabetes medication or insulin, or being currently under treatment for diabetes. PD was defined as a fasting plasma glucose concentration between 100 and 125 mg/dL (5.6–6.9 mmol/L), no use of diabetes medication or insulin, and not being under treatment for diabetes. NGT was defined as a fasting plasma glucose concentration < 100 mg/dL (5.6 mmol/L), with no use of diabetes medication or insulin, and not being under treatment for diabetes.

### 4.4. Assessment of Dietary Intake

Dietary intake data were assessed using a validated 106-item semi-quantitative food frequency questionnaire (SQ-FFQ) [91]. In the KoGES, dietary intake data were collected only at two time points: at baseline (2001–2002) and at the second follow-up (2005–2006). These were the only periods during which the SQ-FFQ was administered as part of the study design, and no additional dietary intake data were collected in subsequent follow-up surveys. Therefore, our study utilized the available dietary intake data from these two-time points, reflecting the original design and data availability of the KoGES.

The SQ-FFQ categorized frequency of consumption into nine levels: never or seldom, once a month, two to three times/month, once to twice a week, three to four times/week, five to six times/week, once a day, twice a day, and three times or more/day. The answer for portion size had three categories: half of a standard serving, one standard serving, and two or more standard servings. For the analysis, individual food consumption was converted to weekly frequencies and multiplied by the reported portion sizes for each food. The intake of fruit, vegetables, red meat, white meat, grains, fish, and dairy was summed up to obtain the total intake for each food group. Among the 106 items recorded in the SQ-FFQ, fruit (persimmons/dried persimmons, tangerines, oriental melons/melons, bananas, pears, apples, oranges, watermelons, peaches/plums, strawberries, grapes, and tomatoes), vegetables (potatoes, sweet potatoes, radish or salted radish, Korean cabbages, spinach, lettuces, perilla leaves, deodeoks/balloon flowers, bean sprouts/mung bean sprouts, brackens/sweet potato stems/taro stems, red pepper leaves/chamnamuls/chwinamuls, crown daisies/leeks/water parsleys, cucumbers, carrots, onions, green peppers, zucchinis, pumpkins, and mushrooms), red meat (roasted pork, pork belly, braised pork, ham/sausage, roasted beef, beef soup, and edible viscera), white meat (chicken), grains (white rice, barley rice, multigrain rice, and mixed grains), fish (raw fish, hairtail, eel, croaker, pollack, frozen pollack, dried pollack, mackerel, saury, spanish mackerel, anchovy, squid, octopus, tuna/canned tuna, fish cake, crab, clam, oyster, shrimp, and salted seafood), and dairy (milk, yogurt, ice cream, and cheese) were assessed using the SQ-FFQ. Finally, dietary intake was classified into three groups (Tertiles 1, 2, and 3). Participants were ranked based on their weekly intake levels for each food group, and the population was divided into three equal-sized groups (tertiles). Tertile 1 represents the third with the lowest intake, Tertile 2 represents the middle third, and Tertile 3 represents the third with the highest intake. In the statistical analyses, Tertile 1 was used as the reference group for comparisons, as presented in Table 1.

For participants who developed PD or T2D or were censored between the baseline and the second follow-up survey, their dietary intake was recorded as the value at baseline. For those who developed PD or T2D or were censored after the second follow-up survey, their dietary intake was calculated as the mean of the values at baseline and the second follow-up.

### 4.5. Genotyping and Quality Control

Genomic DNA from the participants were genotyped using the Korea Biobank Array (KCHIP, KORV 1.1), a customized genotyping platform specifically designed for the Korean population [32]. The KCHIP array was developed by the Center for Genomic Science at the Korea National Institute of Health, based on the UK Biobank Axiom Array, and was manufactured by Affymetrix. This array includes a total of 833,535 SNPs for autosomal chromosomes, and was optimized to capture genetic variations specific to East Asian populations. The genomic locations of the SNPs were assigned according to the human reference genome build hg19 (GRCh37). To ensure the reliability of the genotyping data, quality control procedures were performed using PLINK v1.9.0 (National Institutes of Health, Bethesda, MD, USA) [92]. SNPs with missing call rates over 5%, indicating poor genotyping quality, were excluded from the analysis. Variants with a minor allele frequency (MAF) of less than 1% were also excluded, because rare variants might lack sufficient statistical power for the analysis used in this study. Additionally, SNPs with a Hardy–Weinberg equilibrium (HWE) *p*-value of less than 1.0 × 10^−5^ were removed, to avoid potential genotyping errors or issues related to population stratification. After applying these quality control criteria, a total of 537,149 high-quality SNPs remained and were included in the subsequent analyses.

### 4.6. Statistical Analysis

**Figure 3:**
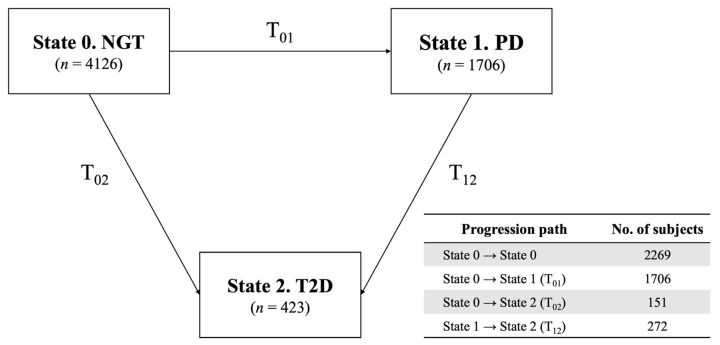
Framework of multi-State illness–death model for type 2 diabetes progression. This figure depicts multi-state illness–death model used to analyze transitions between type 2 diabetes states: normal glucose tolerance (NGT), prediabetes (PD), and type 2 diabetes (T2D). Model includes three transitions: NGT → PD, NGT → T2D, and PD → T2D. Arrows represent possible transitions between states during the follow-up period. Counts of participants transitioning between states are provided alongside each arrow to summarize observed data.

In this study, a ‘multi-state illness–death’ model was applied, which aimed to estimate the probabilities of different transitional states: transition 1: NGT to PD, transition 2: NGT to T2D, and transition 3: PD to T2D. The different risk factors were evaluated for each transition, and a multi-state illness–death model was generated, as described in Figure 3 [26,93]. In this model, subjects moved from state i to state j over time T_ij_; the PD and T2D states were treated as the intermediate and absorbing states, respectively. The factors influencing the hazards for the transitions from NGT to PD (T_01_: transition 1), NGT to T2D (T_02_: transition 2), and PD to T2D (T_12_: transition 3) were investigated. These three transitions could occur at any time until the end of the follow-up.

Cox regression analyses were applied to calculate the transition hazards (α_01_, α_02_, and α_12_). The transition models were fitted to model the hazard for each transition by using the ‘coxph()’ function from the R-package *survival* (version 3.5.8) [94]. Based on the synergistic effects of genetic and lifestyle factors on Cox regression analysis, we proposed a two-stage model to demonstrate the influence of PD and T2D. In the first stage, the risk factors of PD and T2D, including epidemiological variables (age, BMI, sex, residential area, education level, household income, smoking status, alcohol consumption, and physical activity) and dietary intake variables (fruit, vegetable, red meat, white meat, grain, fish, and dairy) were considered in a Cox proportional hazards model. In the next stage, we investigated associations of genetic factors with PD and T2D for each transition using the Cox proportional hazards model, after adjustment for the epidemiological and significant dietary intake variables identified in the previous stage.

The proportional hazards assumption was verified based on Schoenfeld’s residuals [95], by using the ‘cox.zph()’ function from the R-package *survival* (version 3.5.8) [94]. The HRs and 95% CIs were calculated according to dietary intake and SNPs. The *p*-value for each SNP was adjusted for multiple tests using the false discovery rate (FDR) [96]. This adjustment controls for multiple testing by estimating the proportion of false positives among the significant findings. A *q*-value threshold of less than 0.05 was applied, meaning that when a *q*-value was below this threshold, we expected that fewer than 5% of those significant associations were false positives. Statistical analyses were performed using R software version 4.2.2 (R Core Team 2022, Vienna, Austria) [97] and SPSS version 27.0 (IBM Corp., Armonk, NY, USA) [98]. A two-sided *p*-value of less than 0.05 was considered statistically significant.

## 5. Conclusions

This study provides valuable insights into how dietary factors and genetic variants independently influence PD and T2D progressions within the Korean population. Using a multi-state illness–death model, we offer a nuanced interpretation of T2D progression, covering transitions from NGT to PD, NGT to T2D, and PD to T2D. Our findings reveal significant associations between specific dietary patterns and PD/T2D incidence among individuals with NGT. We identify novel genetic variants associated with progression from NGT to PD and NGT to T2D. These genetic markers and dietary factors offer a comprehensive understanding of T2D development. Identifying stage-specific genetic and dietary influences suggests potential for targeted prevention strategies and personalized interventions. Our results emphasize the importance of considering environmental and genetic factors in T2D risk assessment. While our study contributes significantly to the field, further research is needed to validate these findings in diverse populations and elucidate how these factors influence disease progression.

## Figures and Tables

**Table 1:** Hazard ratios (HRs) for transitions between glucose tolerance states, by dietary intake tertiles. This table shows hazard ratios (HRs) and 95% confidence intervals (CIs) for transitions between normal glucose tolerance (NGT), prediabetes (PD), and type 2 diabetes (T2D), based on dietary intake tertiles. Dietary intake was classified into tertiles based on weekly consumption levels for each food group. Tertile 1 represents the lowest-intake group, and is used as the reference category. HRs were adjusted for age, sex, BMI, physical activity, smoking status, alcohol intake, education level, and household income.

Variables	NGT to PD		NGT to T2D		PD to T2D	
	HR (95% CI)	*p*-Value	HR (95% CI)	*p*-Value	HR (95% CI)	*p*-Value
Fruit						
Tertile 1	Ref		Ref		Ref	
Tertile 2	0.27 (0.21–0.34)	<0.001	0.68 (0.46–1.02)	0.063	0.92 (0.68–1.24)	0.570
Tertile 3	0.18 (0.14–0.24)	<0.001	0.60 (0.39–0.93)	0.022	0.99 (0.72–1.36)	0.951
Vegetable						
Tertile 1	Ref		Ref		Ref	
Tertile 2	0.97 (0.85–1.10)	0.601	0.68 (0.45–1.03)	0.067	0.96 (0.69–1.33)	0.810
Tertile 3	1.11 (0.97–1.27)	0.118	0.63 (0.40–1.00)	0.048	1.25 (0.90–1.73)	0.188
Red meat						
Tertile 1	Ref		Ref		Ref	
Tertile 2	1.07 (0.83–1.38)	0.584	0.90 (0.59–1.38)	0.636	1.15 (0.83–1.58)	0.396
Tertile 3	1.41 (1.08–1.85)	0.012	0.88 (0.54–1.45)	0.624	1.16 (0.80–1.67)	0.435
White meat						
Tertile 1	Ref		Ref		Ref	
Tertile 2	1.05 (0.83–1.34)	0.673	1.04 (0.70–1.55)	0.828	0.95 (0.71–1.29)	0.757
Tertile 3	1.61 (1.26–2.07)	<0.001	1.21 (0.77–1.90)	0.415	0.98 (0.71–1.37)	0.925
Grain						
Tertile 1	Ref		Ref		Ref	
Tertile 2	0.95 (0.84–1.07)	0.402	0.96 (0.62–1.47)	0.845	0.90 (0.66–1.23)	0.527
Tertile 3	1.00 (0.88–1.13)	0.971	1.32 (0.88–1.96)	0.177	1.00 (0.74–1.35)	0.992
Fish						
Tertile 1	Ref		Ref		Ref	
Tertile 2	0.86 (0.68–1.10)	0.235	0.98 (0.63–1.53)	0.923	0.88 (0.63–1.23)	0.454
Tertile 3	1.17 (0.91–1.51)	0.217	1.62 (1.00–2.62)	0.048	1.05 (0.74–1.50)	0.770
Dairy						
Tertile 1	Ref		Ref		Ref	
Tertile 2	0.62 (0.49–0.78)	<0.001	1.22 (0.82–1.80)	0.333	0.92 (0.68–1.24)	0.583
Tertile 3	1.02 (0.81–1.28)	0.887	1.12 (0.73–1.74)	0.605	0.92 (0.67–1.26)	0.596

**Table 2:** Characteristics of identified novel variants associated with transitions between glucose tolerance states. This table summarizes characteristics of single-nucleotide polymorphisms (SNPs) identified as significantly associated with transitions between glucose tolerance states: normal glucose tolerance (NGT), prediabetes (PD), and type 2 diabetes (T2D). Table includes information on chromosome number, genomic position, SNP identifiers, reference and alternative alleles, nearest gene, hazard ratios (HRs) with 95% confidence intervals (CIs), *p*-values, and *q*-values. *q*-value is false discovery rate (FDR)-adjusted *p*-value that controls for multiple testing.

Model	Chr ^a^	Pos ^b^	SNP ^c^	Alleles ^d^	Nearest Gene	HR (95% CI) ^e^	*p*-Value	*q*-Value
NGT ^f^ → PD ^g^	7	44235668	*rs4607517*	G/A	*GCK*	1.27 (1.17–1.37)	1.37 × 10^−9^	0.0006
	7	44257943	*rs758982*	C/T	*CAMK2B*	1.27 (1.18–1.38)	2.38 × 10^−8^	0.0006
NGT → T2D ^h^	15	42733571	*rs145386384*	A/G	*ZNF106*	3.77 (2.36–6.00)	2.41 × 10^−8^	0.0123
	19	50360989	*rs59595912*	A/G	*PTOV1*	2.64 (1.85–3.77)	8.22 × 10^−8^	0.0210
	2	83637190	*rs7575023*	T/C	*LOC105374834*	1.88 (1.49–2.38)	1.49 × 10^−7^	0.0253
	13	95600085	*rs35566993*	A/G	*LINC00557*	2.76 (1.86–4.09)	4.74 × 10^−7^	0.0445
	20	17374513	*rs11698919*	A/G	*PCSK2*	2.06 (1.55–2.73)	6.23 × 10^−7^	0.0445
	1	47931749	*rs59813747*	C/T	*FOXD2*	3.66 (2.20–6.10)	6.29 × 10^−7^	0.0445
	16	58700803	*rs4784964*	C/T	*SLC38A7*	3.15 (2.00–4.94)	6.47 × 10^−7^	0.0445
	1	72341074	*rs147467153*	A/G	*NEGR1*	4.31 (2.42–7.67)	6.97 × 10^−7^	0.0445

^a^ chromosome; ^b^ position; ^c^ single-nucleotide polymorphism; ^d^ reference allele/alternative allele; ^e^ hazard ratio (95% confidence interval); ^f^ normal glucose tolerance (NGT); ^g^ prediabetes (PD); ^h^ type 2 diabetes (T2D). Adjusted for age, BMI, sex, residential area, education level, household income, smoking status, alcohol consumption, activity, and food intake variables.

**Table 3:** Functional prediction scores for identified SNPs. Table includes information on chromosome position, SNP identifiers, nearest genes, and two key prediction scores: Combined Annotation-Dependent Depletion (CADD) and Deleterious Annotation using Neural Networks (DANN). Higher CADD and DANN scores indicate greater pathogenic potential of variants. These results highlight the functional impact of genetic variants on disease progression.

Model	Chr ^a^	Pos ^b^	SNP ^c^	Nearest Gene	CADD Score ^d^	DANN Score ^e^
NGT ^f^ PD ^g^	7	44235668	*rs4607517*	*GCK*	14.470	0.877
	7	44257943	*rs758982*	*CAMK2B*	8.908	0.743
NGT T2D ^h^	15	42733571	*rs145386384*	*ZNF106*	5.721	0.715
	19	50360989	*rs59595912*	*PTOV1*	1.083	0.481
	2	83637190	*rs7575023*	*LOC105374834*	0.025	0.573
	13	95600085	*rs35566993*	*LINC00557*	0.695	0.391
	20	17374513	*rs11698919*	*PCSK2*	5.039	0.318
	1	47931749	*rs59813747*	*FOXD2*	12.890	0.994
	16	58700803	*rs4784964*	*SLC38A7*	4.847	0.802
	1	72341074	*rs147467153*	*NEGR1*	0.733	0.481

^a^ chromosome; ^b^ position; ^c^ single-nucleotide polymorphism; ^d^ Combined Annotation-Dependent Depletion score; ^e^ Deleterious Annotation of Genetic Variants using Neural Networks score; ^f^ normal glucose tolerance; ^g^ prediabetes; ^h^ type 2 diabetes.

## Data Availability

The KARE Korean Chip (KORV1.1) datasets are a part of the KoGES, and are available upon approval by the genome center at the Korea National Institute of Health (https://is.kdca.go.kr/ (accessed on 10 March 2025)).

## Supplementary Materials

The following supporting information can be downloaded at https://www.mdpi.com/article/10.3390/ijms26062597/s1.
