# Supplementary material for: Identification of Novel Genetic Variants and Food Intake Factors Associated with Type 2 Diabetes in South Korean Adults, Using an Illness–Death Model
Source: Int J Mol Sci. 2025 Mar 13;26(6):2597. doi: 10.3390/ijms26062597 (PMC11942363; doi:10.3390/ijms26062597)
Supplement: Supplementary file 1 [file ijms-26-02597-s001.zip › ijms-3445978-supplementary.pdf]

Supplementary Tables and Figures

Identification of Novel Genetic Variants and Food Intake Factors Associated with Type 2 Diabetes in South Korean Adults Using an Illness-Death Model

Jeongmin Oh [1], Junho Cha [2] and Sungkyoung Choi [1,2,3*]

[1] Department of Applied Mathematics, College of Science and Convergence Technology, Hanyang University, 55 Hanyang-daehak-ro, Sangnok-gu, Ansan 15588, South Korea; ojm0027@hanyang.ac.kr
[2] Department of Applied Artificial Intelligence, College of Computing, Hanyang University, 55 Hanyang-daehak-ro, Sangnok-gu, Ansan 15588, South Korea; chajunho822@hanyang.ac.kr
[3] Department of Mathematical Data Science, College of Science and Convergence Technology, Hanyang University, 55 Hanyang-daehak-ro, Sangnok-gu, Ansan 15588, South Korea; day0413@hanyang.ac.kr
* Correspondence: day0413@hanyang.ac.kr; Tel.: +82-31-400-5465

Table S1. Baseline characteristics of the study participants (2001-2002). Variables include demographic factors (age, sex, education level), lifestyle factors (smoking status, alcohol intake, physical activity), clinical measurements (BMI, fasting glucose levels), and dietary intake (total energy intake and food group consumption). Continuous variables are expressed as mean ± standard deviation (SD), while categorical variables are presented as counts and percentages.

**Characteristics**	***N* = 4,126**
**Age, year, mean (SD)**	51.0 (8.4)
Survival time for transition 1 (NGT to PD), years, median (inter-quantile range)	5.8 (2.0–7.9)
Survival time for transition 2 (NGT to T2D), years, median (inter-quantile range)	13.8 (13.7–14.0)
Survival time for transition 3 (PD to T2D), years, median (inter-quantile range)	3.45 (1.8–5.7)
**Follow-up time, years, median (inter-quantile range)**	13.8 (13.6–14.0)
**Sex, no (%)**	
Males	1,943 (47.1)
Females	2,183 (52.9)
**Residential area, no (%)**	
Ansan	2,321 (56.3)
Ansung	1,805 (43.7)
**Physical activity, no (%)**	
No	1,300 (31.5)
Yes	2,826 (68.5)
**Alcohol intake, no (%)**	
Never	1,871 (45.3)
Past	243 (5.9)
Current	2,012 (48.8)
**Smoking status, no (%)**	
Never	2,510 (60.8)
Past	654 (15.9)
Current	962 (23.3)
**Education level, no (%)**	
Elementary	1,167 (28.3)
Middle/high	2,361 (57.2)
College/higher	598 (14.5)
**Household income (Korean won/month), no (%)**	
< 1 million	1,240 (30.1)
1 to < 2 million	1,251 (30.3)
2 to < 3 million	819 (19.8)
≥ 3 million	816 (19.8)
**BMI, kg/m^2, mean (SD)**	24.5 (3.0)
**Food intake (serving/week), median (inter-quantile range)**	
Fruit	12.3 (4.4–14.1)
Vegetable	26.6 (14.6–33.4)
Red meat	2.8 (1.1–3.6)
White meat	0.8 (0.2–0.9)
Grain	25.6 (22.7–26.4)
Fish	8.8 (3.7–11.8)
Dairy	5.2 (0.8–7.8)

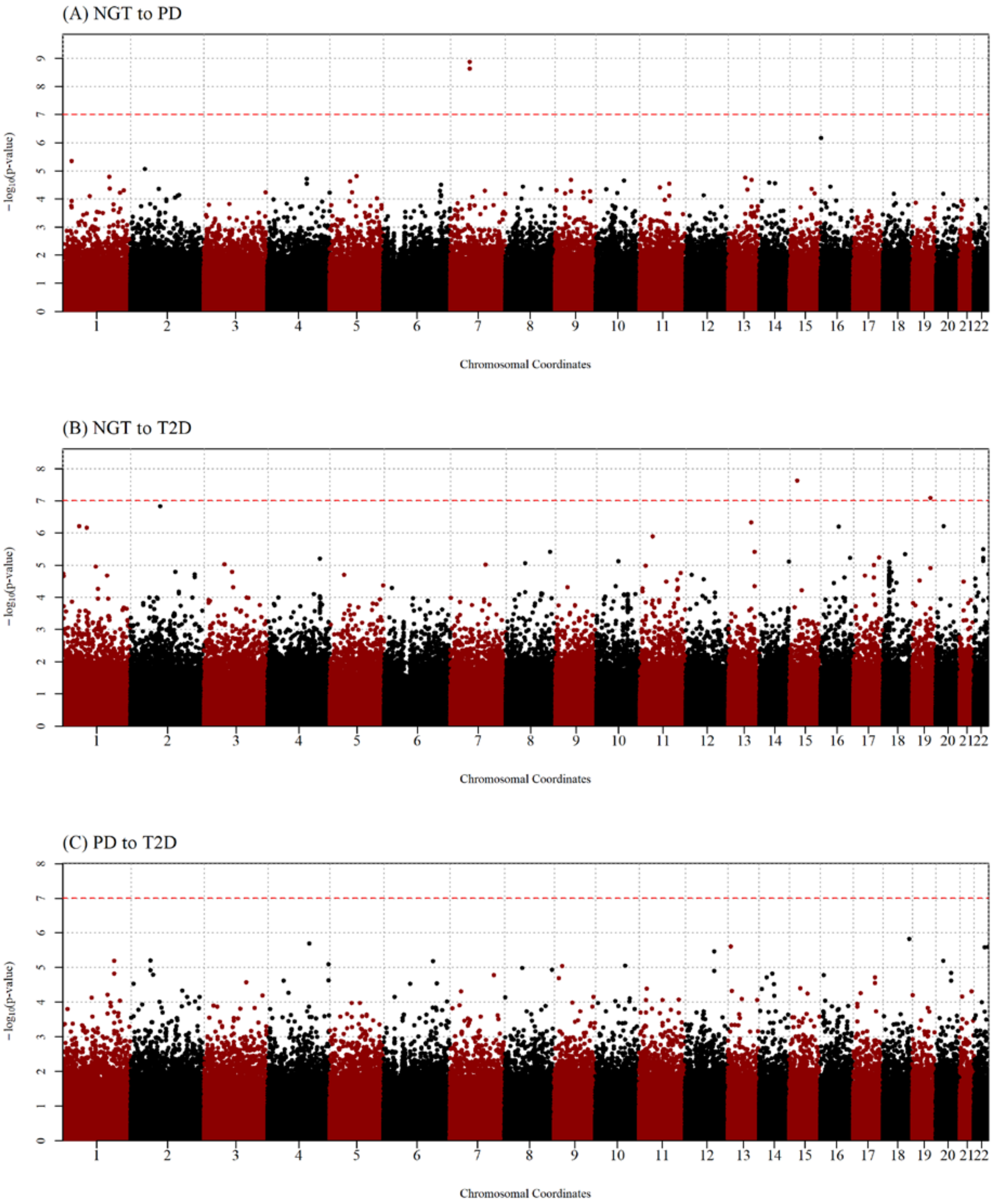

Figure S1. Manhattan plots of genome-wide association study results for three different transition models. (A) NGT to PD model (B) NGT to T2D model. (C) PD to T2D model. Each genetic variant is indicated by a dark-red or black dot. The *y*-axis represents the -log10(*p*-value). The *x*-axis represents chromosomal positions. The dashed red horizontal line indicates the genome-wide significance threshold at *p*-value < 1×10^{-8}. Normal glucose tolerance (NGT), Prediabetes (PD), Type 2 diabetes (T2D).

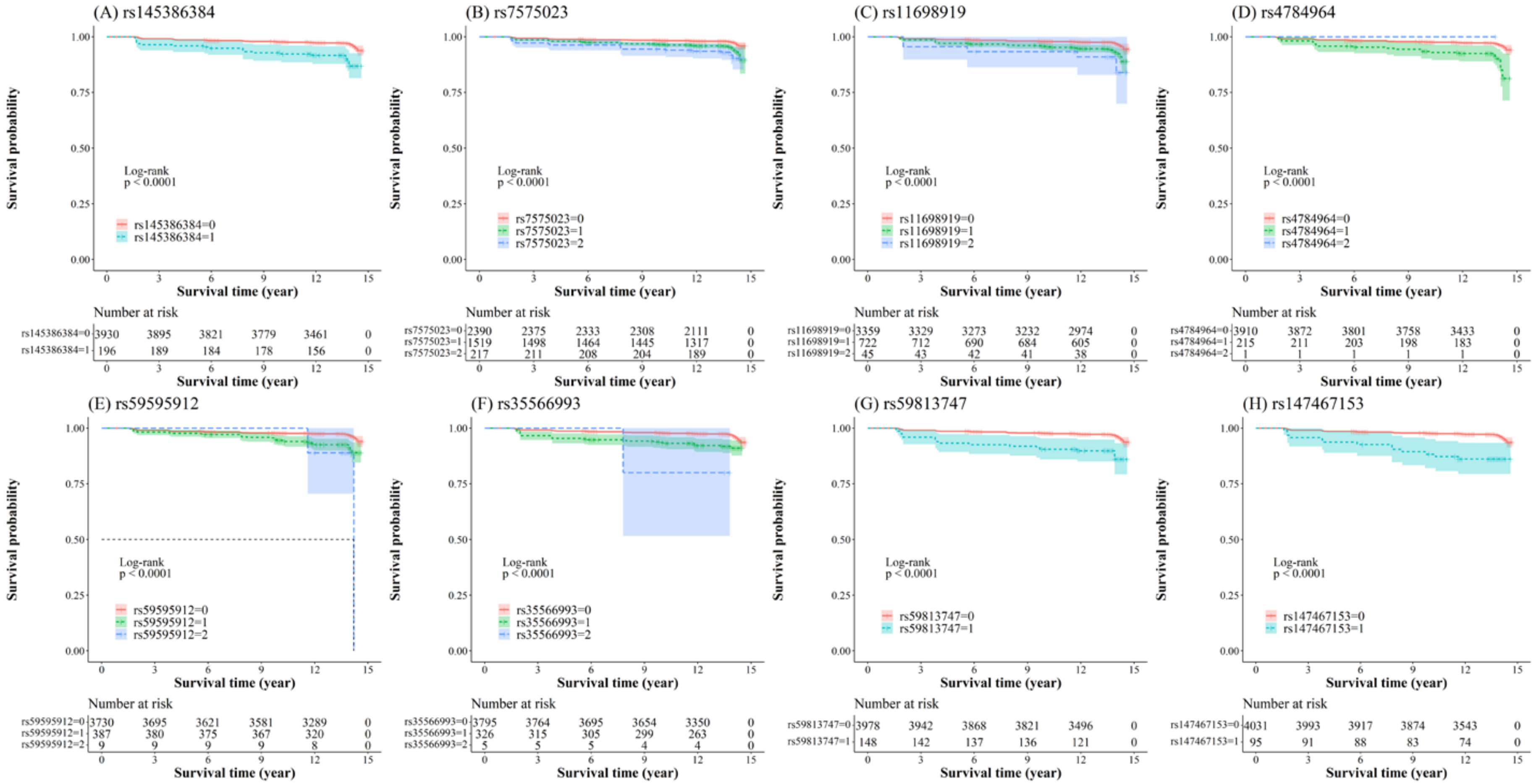

Figure S2. The Kaplan-Meier survival curves illustrate the cumulative risk of T2D in the NGT to T2D model based on 0 (upper red line), 1 (middle green line), and 2 (lower blue line) allele counts of SNPs. (A) Survival curve for *rs145386384*. (B) Survival curve for *rs7575023*. (C) Survival curve for *rs11698919*. (D) Survival curve for *rs4784964*. (E) Survival curve for *rs59595912*. (F) Survival curve for *rs35566993*. (G) Survival curve for *rs59813747*. (H) Survival curve for *rs147467153*. *p*-value < 0.0001 from log-rank test. Normal glucose tolerance (NGT), Prediabetes (PD), Type 2 diabetes (T2D).

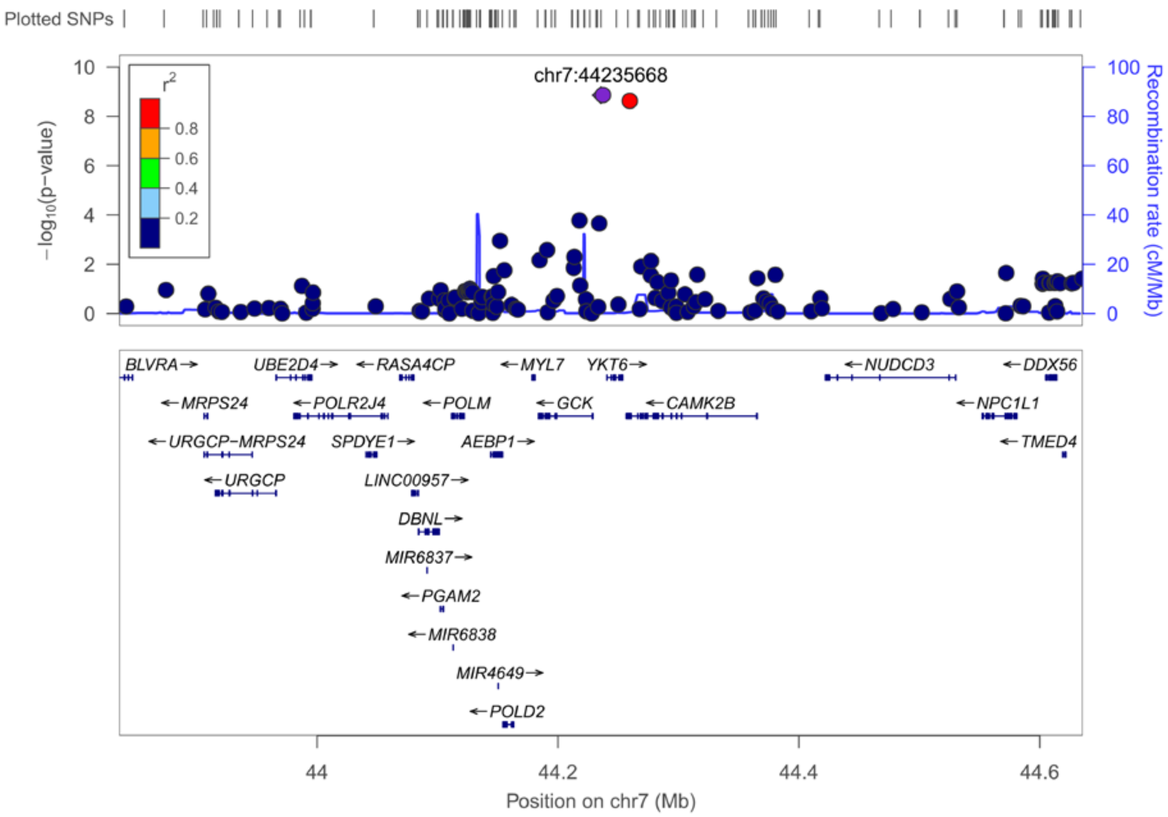

Figure S3. Regional plot of *rs4607517* that was significantly discovered from the NGT to PD model. The SNPs shown in the figure are located on chromosome 7:43835668–44635668. The strongest significant SNP, *rs4607517*, was shown in purple, and the red dot represents chromosomal positions of *rs758982* near the *rs4607517*.
